# Performance of Artificial Diets for *Zelus renardii* (Hemiptera: Reduviidae) Rearing

**DOI:** 10.3390/insects15080607

**Published:** 2024-08-12

**Authors:** Ugo Picciotti, Miguel Valverde-Urrea, Valdete Sefa, Marco Ragni, Francesca Garganese, Francesco Porcelli

**Affiliations:** 1 Dipartimento di Scienze del Suolo, della Pianta e degli Alimenti, University of Bari Aldo Moro, 70126 Bari, Italy; ugo.picciotti@uniba.it (U.P.); valdetesefa@gmail.com (V.S.); marco.ragni@uniba.it (M.R.); francesco.porcelli@uniba.it (F.P.); 2 Laboratory of Plant Pathology, Department of Marine Sciences and Applied Biology, University of Alicante, 03080 Alicante, Spain; mrvu1@gcloud.ua.es

**Keywords:** alien invasive, olive quick decline syndrome, biofactory, IPM

## Abstract

**Simple Summary:**

*Zelus renardii* Kolenati (Hemiptera, Reduviidae, Harpactorinae), the Leafhopper Assassin Bug (LAB), first appeared in Europe in 2011 and is now well-acclimatized. The LAB is a promising mass-rearing candidate for the inundative biocontrol strategy of *Xylella fastidiosa pauca* ST53 vectors. We reared LABs for two subsequent years, using live adult *Drosophila melanogaster* Meigen (Diptera, Drosophilidae) (Dm) as living prey versus several artificial diet formulations (D0, D1, D2, D3, and D4), to identify the best formulation. An artificial medium can minimize rearing costs and make the process sustainable and safer. The rearing of LAB is feasible with live prey, oligidic, meridic, and holidic artificial formulations. All diets, except for D2, showed a favorable trend according to the species’ reproductive potential. Scoring accumulated degree days in LAB’s rearing made it possible to predict the time required to complete post-embryonic development as a function of temperature.

**Abstract:**

Mass production is a prerequisite for using natural enemies in integrated pest management and organic farming. Natural enemies in agroecosystems include predators that prey on insects, which they can subdue while maintaining adequate pest population densities. The Leafhopper Assassin Bug (LAB), *Zelus renardii*, can be a natural enemy in agroecosystems, selecting its prey for size and mobility. Some of LAB’s prey include *Philaenus spumarius* (L.), *Bactrocera oleae* (Rossi), *Drosophila suzukii* (Matsumura), and *Macrohomotoma gladiata* Kuwayama, suggesting this reduviid for biocontrol agent in various contexts. We reared LABs for two subsequent broods offering living prey and artificial diets. Our data show that the rearing of *Z. renardii* is feasible with oligidic, meridic, and holidic artificial formulations. Four artificial diets allowed the complete post-embryonic development of LABs in captivity for two successive generations. The accumulated degree-days (ADDs) accurately predict the growth of LABs based on heat accumulation, estimating that up to three generations could grow per year in captivity at the experimented T°C.

## 1. Introduction

Worldwide, more than 230 naturally antagonistic arthropod species [1] are reared, mostly parasitoid and predator insects [2]. The mass-rearing of insects involves operations to increase the reproductive potential, or fecundity, of the reared species [3,4]. Mass-rearing aims to produce many healthy and reproductive individuals [5]. Mass-rearing is a prerequisite for using natural enemies in IPM and organic farming [6] for inundative and different biocontrol actions. There is a growing interest in rearing insects in biofarms due to using these arthropods in food and feed production [7,8].

Rearing systems are simplified microcosms acting as artificial niches, providing optimal conditions for successful mass-rearing [9,10]. Insect biofactories produce fertile and voracious genetically stable individuals, i.e., capable of transmitting selected traits to their offspring [11]. Mass-rearing requires control of temperature, photoperiod, and feeding [11]. The diet can consist of live prey for predators, hosts for parasitoids, or complex artificial media. The most common practice is rearing natural enemies on live insects; however, this is demanding in cost and labor and is often unsustainable. Furthermore, using live insects poses health problems by exposing them to epizootics (e.g., entomopathogens, such as fungi, viruses, or bacteria) [12].

An artificial diet usually reduces rearing costs, making it sustainable and safer. Bogdanow [13] first started developing artificial diets for insects, leading to modern formulations. The diet formulation must adapt to the needs of the antagonist, adjusting the fraction of acquirable nutrients depending on the species and instars. In nature, nutrient availability limits feed quantity and quality, shaping the antagonist’s feeding habits [10]. The composition of artificial diets can be chemically defined (holidic), chemically semi-defined (meridic), or chemically undefined (oligidic) [14,15]. The components of a diet must meet the requirements of palatability, nutritional completeness (ratio of macro- to micronutrients), stability (chemical, physical, and biological), and insect bioavailability [10] for each antagonist entity.

Natural enemies in agroecosystems include predators [16] that prey on insects that they can perceive for preying and subdue while maintaining adequate pest population densities [17]. Almost all insect orders [18] have predatory species, of which predatory hemipterans contribute most to the natural and applied biological control of pests [19,20]. Most predatory hemipterans belong to the Geocoridae, Anthocoridae, Nabidae, Pentatomidae, and Reduviidae genera [17]. Several hemipterans thrive on natural or artificial diets [17,21,22,23,24,25,26,27]. Reared individuals or cohorts of *Rhynocoris* spp., *Platymeris* spp., and *Zelus* spp. [27] serve as natural enemies in agroecosystems.

*Zelus* is one of the Reduviidae genera, comprising 71 species [28,29] native to Tropical and Subtropical America [30,31]. Globalization and trade in fresh crops have allowed *Zelus renardii*, the Leafhopper Assassin Bug (LAB), to enter and spread in several European and Mediterranean countries, where it has acclimated [32,33,34,35,36,37,38,39,40,41,42,43,44,45,46].

*Zelus renardii* is a stenophagous predator [47], selecting its prey based on size and mobility [47]. LAB uses visual stimuli to direct the movement of potential prey through ambushing, stalking, or entangling [48,49,50,51]. LAB preys on pests, including *Bactrocera oleae*, *Bactrocera cucurbitae* (Coquillett) (Diptera, Tephritidae), *Ricania shantungensis* Chou and Lu (Hemiptera, Ricaniidae), and *Philaenus spumarius* (Hemiptera, Aphrophoridae), of which the latter is a vector of *Xylella fastidiosa* Wells et al. (Xf) subsp. *pauca* ST53 [52,53,54,55,56,57,58]. The Xf subsp. *pauca* ST53 in Italy is responsible for the Olive Quick Decline Syndrome (OQDS), which can lead to the death of olive trees in a few years. The OQDS epidemic [52,59] has incentivized the study of the control of Xf candidates or vectors in the Mediterranean. *Xylella fastidiosa* has an unprecedented agroecological, economic, social, and political impact among most plant pathogens [60]. Multidisciplinary approaches drive the search for a strategy to manage the epidemic and transmission [61,62,63].

Various studies have focused on identifying the autochthonous natural enemies of *P. spumarius*. The action of some natural enemies, such as *Ooctonus vulgatus* Haliday (Hymenoptera, Mymaridae) [64], *Verrallia aucta* (Fallén) (Diptera, Pipunculidae) [65], and some spiders [66,67,68], is not effective or in time to manage *P. spumarius* populations, which can range from three to six million individuals per hectare [69]. To date, no native predators specialized in *Xylella* vectors are available. It has been shown that generalist predators, such as spiders, influence the control of certain economically important pests or can, with their webs, form physical barriers to the spread of conidia of some plant pathogens [70]. Although spiders prey on *P. spumarius*, their action cannot contain Xf transmissions [66]. Evidence suggests that LABs may be a promising biocontrol agent in olive grove IPM, as indicated in the cotton fields [49,50,71], and sold as eggs [72,73,74]. Numerical experiments have demonstrated the potential efficacy of the inundative control strategy with LABs against Xf vectors [75]. Biocontrol with LABs could stop new infections and reduce Xf transmission to a manageable threshold [69,75]. Therefore, the use of LAB adults for biological control of Xf infections depends on the rearing of the predator. Furthermore, LAB preys on invasive pests that recently entered Europe (Italy and Spain), such as *Macrohomotoma gladiata* (Hemiptera, Homotomidae) and *Drosophila suzukii* (Diptera, Drosophilidae) [76,77,78] in Apulia, Italy.

We tested live adults of *Drosophila melanogaster* (Dm) vs. artificial diet formulations to select optimal diets for rearing *Z. renardii*.

## 2. Materials and Methods

### 2.1. Insect Sources

Feral adult LABs were collected from citrus trees hosting a mixed infestation of *Aleurocanthus spiniferus* (Quaintance) and *Aleurothrixus floccosus* (Maskell) (Hemiptera, Aleyrodidae) [79,80,81], both non-prey, in the “Ernesto Quagliariello” Campus of the University of Bari Aldo Moro (N 41°06′37″; E 16°52′58″), Bari, Italy, in May 2020. After capture, we used Zhang et al. [29] to identify *Z. renardii* specimens. Then LABs thrived with *D. melanogaster* Oregon-R strain in one Petri dish each to exclude cannibalism, otherwise frequent within different instars. Eight feral pairs mated randomly in the laboratory, giving the first cohort. We picked 198 newborns in the first cohort, populating six treatments. Random pairing adults from the first cohort populated the five treatments in the second cohort. The pairs of parents reared on one type of diet generated the newborns that populated the cohorts subjected to the same diet for the second cohort. All treatments were repeated in the second year (Dm, D0, D1, D3, D4) except D2.

**Figure 1:**
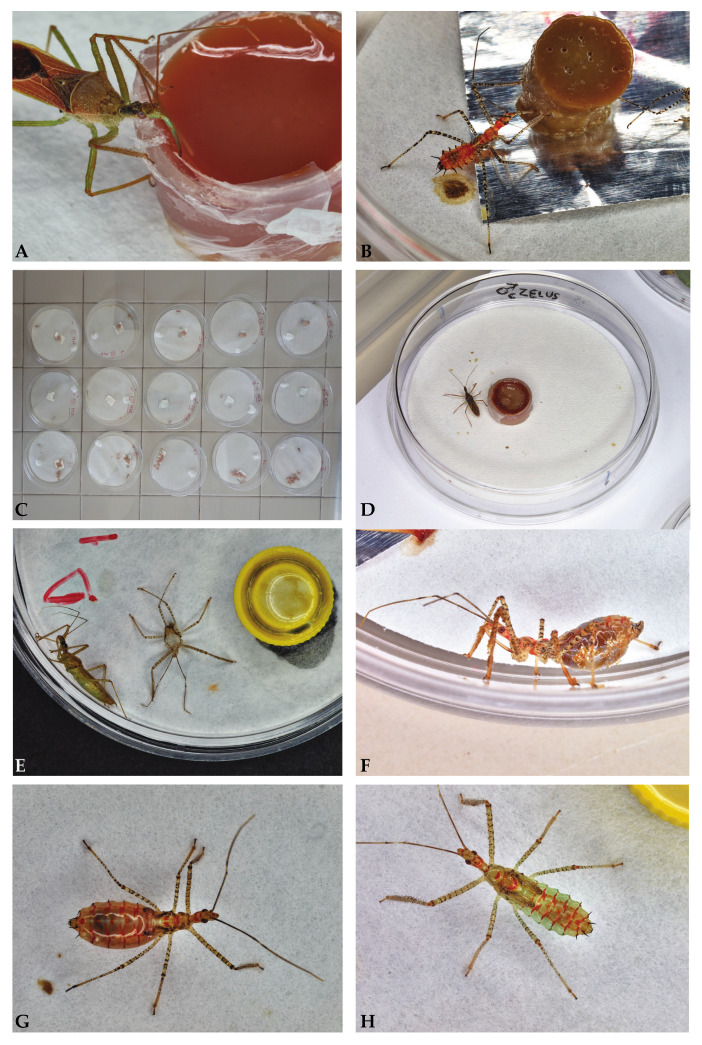
(**A**) Feral adult of *Zelus renardii* accepting D0 liquid formulation; (**B**) *Zelus renardii* third juvenile (N3) feeding on gelled D0; (**C**) *Zelus renardii* cohort rearing in the laboratory; (**D**) adult male of *Zelus renardii* thrived on D0; (**E**) eclosed teneral *Zelus renardii*, reared on D1; (**F**) *Zelus renardii* side view of fourth juvenile (N4), D0-engorged; (**G**) dorsal view of a D3-engorged fourth juvenile (N4) *Zelus renardii*; (**H**) *Zelus renardii* dorsal view of a fasting fifth juvenile (N5).

Each cohort (33 individuals/treatment/generation) originated randomly from pools of eggs laid by females, and the newborns were isolated in vented 3 cm ⦰ Petri dishes 24 h later. We considered each LAB a replicate (363 individuals for both trial generations). Each cohort used a single diet throughout the life cycle (Figure 1C). The five immature instars in the post-embryonic development of the LABs [82] were numbered from N1 to N5, plus AD for adults. Filter paper disks soaked in sterile distilled water maintained the RH at around 80 ± 5%. Additionally, the paper roof of the disks provided a good grip for walking and molting.

The laboratory conditions varied seasonally, with a minimum temperature of 18 °C and a maximum temperature of 25 °C, using air conditioning in the summer and heating in the winter. Photoperiod follows the seasonal variation. Weekly replacing the Petri dishes reduced the contact of LABs with contaminants, mitigating and avoiding epizootics, and daily cleaning removed debris and feces, as reported by Liccardo et al. [75].

### 2.2. Diet Delivery

The artificial diets were initially liquids to test the feral LABs’ acceptance. Aliquots of 0.25 mL of the liquid diets were poured into an Eppendorf microtube cap and delivered to the 9 cm ⦰ Petri dishes (Figure 1A). We gelled the liquid diets with technical agar (Sigma-Aldrich, Merck KGaA, Darmstadt, Germany) after their acceptance. We obtained cylinders of jellified diet by punching the gel with plastic cocktail straws. N1 and N2 ate on cylinders that were 0.2 ⦰ × 1 cm (0.03 cm^3^) in size, carved by 1 mL Pasteur pipettes (Kertell S.p.a., Noviglio, Milan, Italy); while N3, N4, N5, and adults accessed cylinders that were 0.5 ⦰ × 1 cm (0.2 cm^3^) carved by 3 mL Pasteur pipettes (Kertell S.p.a., Noviglio, Milan, Italy) (Figure 1B). A small foil of alloy or PVC (polyvinylchloride) avoided direct contact between diet and filter paper, improving cleanliness and mitigating contamination. Feral adults immediately accepted the liquid formulation of the diets. Based on this response, we chose the formulations to feed the newborns.

### 2.3. Dm: Living Prey, Drosophila Melanogaster

Rearing the LABs with live prey required a continuous supply of adult *D. melanogaster* (vinegar fly—Dm). We supplied five Dm—Oregon-R strain—adults each other day by mouth aspirator to transfer the flies from their flasks to each LAB Petri dish, removing the carcasses. The vinegar flies thrived in 4 L Plexiglas flasks on an artificial meridic medium consisting of 58.9 g of sucrose (Sigma-Aldrich, Merck KGaA, Darmstadt, Germany), 58.9 g of maize meal (Bonomelli s.r.l., Zola Predosa, Itay), 2.5 g of agar (Sigma-Aldrich, Merck KGaA, Darmstadt, Germany), 50 g of fresh brewer’s yeast (Lievital^®^, Lesaffre Italia S.p.A., Trecasali, Italy), 0.5 g of methyl 4-hydroxybenzoate (methylparaben) (Sigma-Aldrich, Merck KGaA, Darmstadt, Germany), and 1.7 mL of propionic acid (Sigma-Aldrich, Merck KGaA, Darmstadt, Germany) per liter of sterile distilled water.

The vinegar flies nourished two cohorts of 33 LABs for two generations and their entire life span.

### 2.4. D0: Beef-Liver–Egg-Yolk–Based Oligidic Diet

The D0 diet had an oligidic base (OB) obtained from 200 g of fresh, homogenized organic beef liver, 20 g of organic egg yolk, and 30 mL of a 30% (*w*/*v*) sucrose solution. The D0 formulation consisted of 50 g of OB, 1 g of agar, 1 g of ascorbic acid (antioxidant) (Sigma-Aldrich, Merck KGaA, Darmstadt, Germany), and 0.5 g of methyl 4-hydroxybenzoate in 100 mL of sterile distilled water.

### 2.5. D1: Holidic Diet Based on Meritene MOBILIS^®^

Meritene MOBILIS^®^ is a nutritionally complete food supplement with a chemically defined composition of carbohydrates, proteins, lipids, minerals, and vitamins. The D1 formulation comprised 20 g of Meritene MOBILIS^®^ (Nestlé Health Science, Nestlé S.A., Vevey, Switzerland), 1 g of agar, 1 g of ascorbic acid, and 0.5 g of methyl 4-hydroxybenzoate in 100 mL of sterile distilled water. The jar of Meritene MOBILIS^®^ reports its composition (Table S1).

### 2.6. D2: Holidic Diet Based on Meritene MOBILIS^®^ and KCl

The D2 formulation corresponded to the D1 formulation by adding potassium chloride (KCl). KCl increased the K content of the artificial diet according to the element’s physiological importance. Moreover, K and Na are indispensable in the reactions of excitable tissues (e.g., nervous tissue), and potassium is also involved in pH regulation in cells and body fluids [10].

The D2 formulation comprised 20 g of Meritene MOBILIS^®^, 0.3 g of KCl (Sigma-Aldrich, Merck KGaA, Darmstadt, Germany), 1.5 g of agar, 1 g of ascorbic acid, and 0.5 g of methyl 4-hydroxybenzoate in 100 mL of sterile distilled water.

### 2.7. D3: Holidic Artificial Diet Based on Nidina^®^ 2 OPTIPRO^®^

Diet D3 is based on Nidina^®^ 2 OPTIPRO^®^ (Nestlé Baby&Me, Nestlé S.A., Vevey, Switzerland), which is a powdered skimmed milk baby food with a balanced supply of protein, vitamins, and minerals. To obtain the D3 formulation, 20 g of Nidina^®^ 2 OPTIPRO^®^, 1 g of agar, 1 g of ascorbic acid, and 0.5 g of methyl 4-hydroxybenzoate were added to 100 mL of distilled water. The jar reports the composition of Nidina^®^ 2 OPTIPRO^®^ (Table S2).

### 2.8. D4: Meridic Artificial Diet Based on Meritene MOBILIS^®^, KCl, and OB

The D4 formulation included 20 g of Meritene MOBILIS^®^ plus 1 g of agar, 10 g of OB, 0.3 g of KCl, 1 g of ascorbic acid, 0.5 g of methyl 4-hydroxybenzoate, and 100 mL of distilled water. As with the other diets, D4 was gelled and distributed in cylinders of different sizes depending on the predator’s instar.

### 2.9. Management of Rearing Data and Pictures

A FileMaker^®^ (FileMaker Inc., Santa Clara, CA, USA), purposely set up as the multimedia database [83], was running on an iPad (Apple Inc., Cupertino, CA, USA) tablet. The database stored all relevant biological rearing events, including hatching, molting, metamorphosis, diet type, sex of adult, date of death, and apparent cause of death.

Several Olympus PEN cameras (Olympus Corporation, Tokyo, Japan) mounted on a Zeiss Tessovar macro/microsystem (Carl Zeiss GA, Oberkochen, Germany) recorded picture shots (.ORF format). All experiments occurred in the Forensic Entomology Laboratory of the DiSSPA–UNIBA Aldo Moro.

### 2.10. Data Analysis

The rearing procedure (1st and 2nd generations) lasted about two years in mean laboratory conditions, as described by Liccardo et al. [75], with a reasonable temperature seasonal cycle [84]. The accumulated degree-days (ADDs) method measured the life cycle length of the LABs based on the different diets. In lifetables, we only considered the data from juveniles of the first egg batch to minimize the influence of the overwintering approximation in the subsequent cohorts. Hoping that such data provided a less biased interval for diet evaluation on insect growth. We consider the T_2_/T_1_ for the first egg batches as the less biased ratio available in the experiments. Conversely, we admit that the development of the second and subsequent cohorts of juveniles may be biased by the overwintering approximation.

Insects are ectothermic, poikilothermic organisms whose body temperature and metabolism change primarily according to environmental temperatures [10]. Thus, the speed of insect development primarily depends on temperature [85]. According to the formula reported by McMaster and Wilhelm [86], we used ADDs to measure accumulated heat units over time as follows:

$$ADD=\sum_{n}^{1}days\left[\frac{\left(Tmax+Tmin\right)}{2}\right]-Tbase$$

where *n* is the number of rearing days, *Tmax* and *Tmin* are the daily maximum and minimum temperatures, and *Tbase* is the development threshold in *Z. renardii*, which is 15 °C [87].

At the end of each generation, we analyzed the sex ratio in each diet with the χ^2^ test, followed by a post hoc analysis based on residuals of Pearson’s Chi-squared Test for Count Data [88]. We analyzed each diet’s mortality with Fisher’s exact test followed by a post hoc test of pairwise comparisons between proportions. In both cases, we applied the Bonferroni *p*-value correction in the multiple comparisons to reduce the probability of making a type I error.

To analyze the effect of diet on ADD accumulation up to the adult stage, we created a new variable (cumulative ADD) by summing the ADD of each instar up to the adult one of every individual. Next, we analyzed variance (ANOVA) with Welch’s correction due to the lack of homogeneity of variances. If significant differences were found, we performed a Games–Howell test, which does not assume equal variances [89].

A Kruskal–Wallis test studied the effect of diet on ADD at each insect instar. Due to the lack of homogeneity of variances, the assumption of a normal distribution of residuals was not met. We considered the diet as a fixed factor.

Moreover, we analyzed the number of laid eggs and their hatching rate using ANOVA and the diet as a fixed factor. We tested the data’s normality distribution with the Lilliefors test on the models’ residuals. Indeed, we used the Levene test for the homogeneity of variances.

For each generation, we performed all the analyses mentioned above.

In addition, we performed a predictive model. A machine learning technique, regression random forest (randomForest package [90]), predicted the ADD of each instar. In regression, random forest, insect instar, and diet were predictor variables. We calculated the optimal number of trees and plotted the results using the Plotmo package [91]. We used the data of the first and second generations for the random forest model.

Finally, we performed a K-means clustering to group diets by similarity following the ADD accumulated at each stage (mean of the two generations), mortality, sex ratio, hatching rate, and eggs per female. Furthermore, we standardized the data by calculating the number of clusters with the Gap statistic method. We used the factoextra package [92] and cluster package [93].

R software v 4.2.2 [94] performed all plots and statistical analyses.

### 2.11. Life Tables

Life tables allow us to assess the actuarial properties of an insect species by analyzing the causes of mortality to know the population trend (*T*) by dividing the number of individuals (eggs) in the subsequent brood over the number in the previous one. The population increases when T_2_/T_1_ > 1 increases, while T_2_/T_1_ < 1 decreases. If T = 1, the population is in equilibrium; usually, this situation is only found in populations in natural ecosystems. We used life tables to quantify the effectiveness of different diet formulations on the LAB-tested cohorts. We used the life tables [95] as a model for understanding the dynamics of LAB cohorts by organizing age-specific mortality and survival data under laboratory conditions. We reported age-specific mortality rates for the life tables during the experimental period. Life expectancy for each stage is calculated in ADD, and all causes of death are grouped by instar. The life tables consider cohort survival (*lx*), age-specific mortality (*qx*), age-specific survival (*px*), and distribution of deaths (*dx*) as main functions, as reported in [95]. Life expectancy per instar (*ex*) is the average ADD value per survived individual.

We estimated *T* since a LAB female lays the average number of eggs after random mating in our rearing system per treatment. The estimation considered the number of females obtained per generation (T_1_ and T_2_) and the average per treatment (T_Dx_).

## 3. Results

### 3.1. Sex and Mortality Ratios

**Figure 2:**
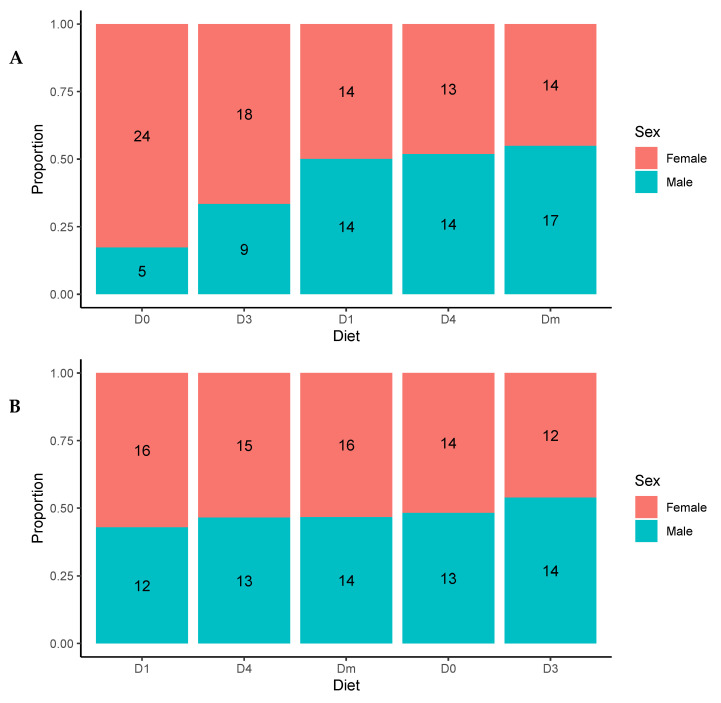
Sex ratio of (**A**) first-generation and (**B**) second-generation laboratory-reared *Zelus renardii* (diets ranked in ascending order of male ratio).

In the first generation, diets were associated with sex composition (χ^2^ = 12.04, df = 4, *p* = 0.016) (Figure 2A). In Dm and D4, the proportion of males was higher, while in D1, the balance of both sexes was equal. Furthermore, D3 and D0 showed more females, although this composition was only significant in D0. This relationship was not found in the second generation (χ^2^ = 0.68, df = 4, *p* = 0.95) (Figure 2B).

**Figure 3:**
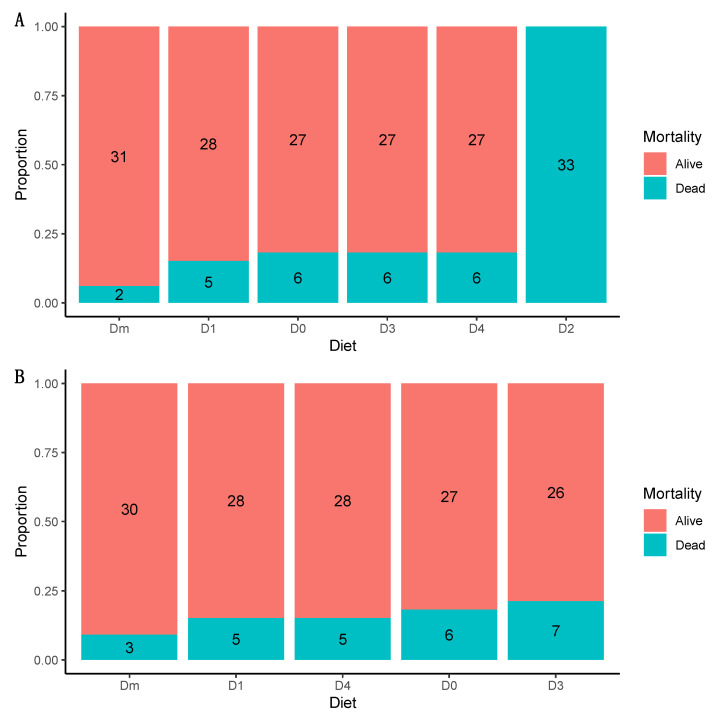
The mortality rate of (**A**) first-generation and (**B**) second-generation laboratory-reared *Zelus renardii* (diets ranked in ascending order of mortality).

D2 showed the highest mortality, with 100% mortality (Figure 3A) in the first generation. Only D2 mortality, compared to the rest of the other diets, showed significant differences (*p* < 0.001). Given these results, we excluded D2 for the tests on the second generation. However, Dm had the lowest mortality in the first generation. In the second generation, there were no significant differences in mortality between diets (*p* = 0.96) (Figure 3B).

### 3.2. Effect of Diet on the ADDs

**Figure 4:**
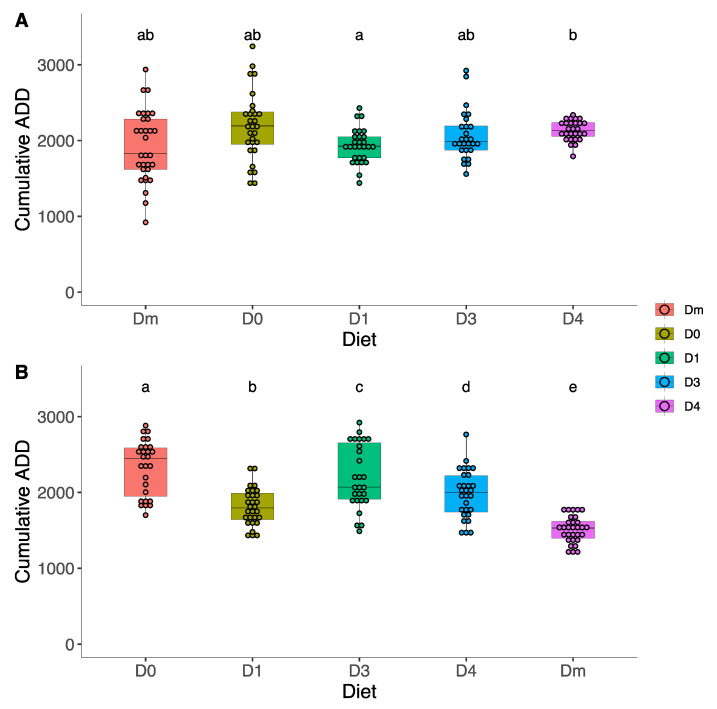
Effect of D0–D1–D3–D4–Dm diets in cumulative ADD to complete *Zelus renardii* post-embryonic development in the first generation (**A**) and second generation (**B**). Lowercase letters indicate significant difference between diets.

In the first generation, D4 showed the highest cumulative ADD (2191.98 ± 448.72 ADD), followed by diet D3 (2131.81 ± 321.27 ADD) (Figure 4A). Conversely, Dm showed the lowest value (1923.96 ± 466.98 ADD), closely followed by D1 (1931.89 ± 255.40 ADD). Welch’s ANOVA found significant differences in cumulative ADD (F_4,62.29_ = 5.16, *p* = 0.001) (Table S3), although the Games–Howell post hoc test only found significant differences between D1 and D4. Note the greater dispersion of data in Dm and D0 compared to the other diets.

In the second generation (Figure 4B), D0 showed the highest cumulative ADD (2332 ± 361.10 ADD), followed by D3 (2203 ± 419.54 ADD). Also, in this case, the Dm value was the lowest (1513 ± 175.99 ADD), followed by the D1 value (1810 ± 243.49 ADD). Cumulative ADD was statistically different between all studied diets (F_4,65.5_ = 41.70, *p* < 0.001) (Table S4). In the second generation, the D0 and D3 presented the highest dispersion of data.

### 3.3. Effect of Diet on Each Instar

**Figure 5:**
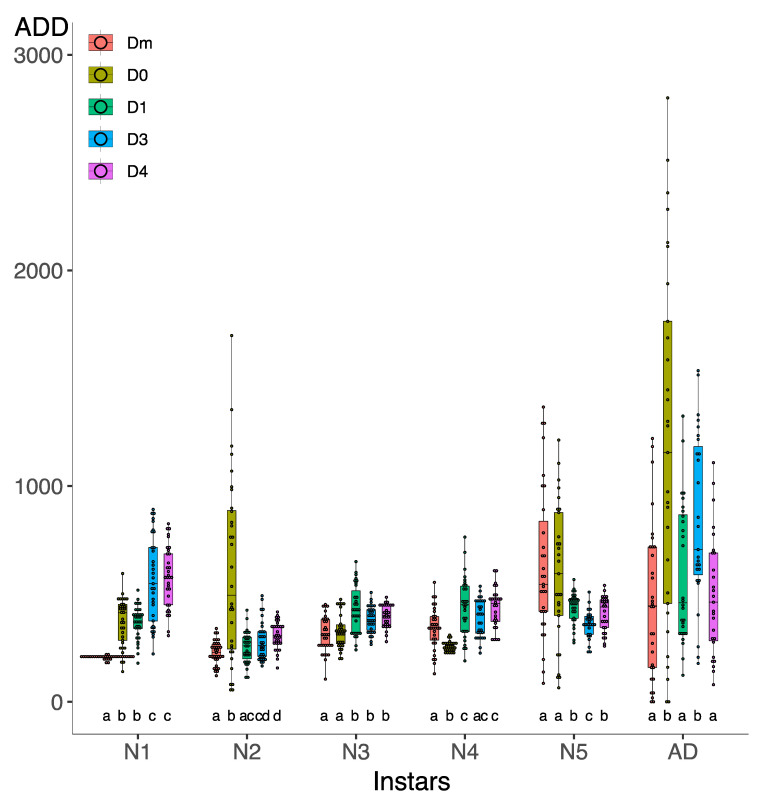
Effect of D0–D1–D3–D4–Dm diets on ADD for each *Zelus renardii* post-embryonic instar in the first generation. Abbreviations: N1 = first instar; N2 = second instar; N3 = third instar; N4 = fourth instar; N5 = fifth instar; AD = adult; ADDs = accumulated degree-days. Lowercase letters indicate significant difference between diets in each instar.

In the first generation, Kruskal–Wallis analysis of cumulative ADD per instar showed significant differences between diets at each instar (Figure 5). In the N1 instar, all artificial diets showed higher ADD than Dm. Conversely, D3 and D4 led to a significantly higher ADD value than D0 and D1.

In the N2 instar, the differences in ADD between the natural diet Dm and D0/D1 were non-significant, but they ranked between Dm and the rest of the diets. Furthermore, in the N3 instar, the D0 and Dm did not show significant differences. However, Dm and D0 have lower ADD than all the other artificial diets.

For the N4 instar, there were no significant differences between Dm and D3, but all the other diets accumulated more ADD than Dm. In the case of N5, Dm and D0 showed similar ADD, and Dm/D0 ADD was significantly lower than that of the other diets.

Finally, Dm, D1, and D4 have significantly lower ADD values for adults than for the rest of the diets. The high dispersion of data in the N2, N5, and AD of artificial diet D0 is noteworthy. Furthermore, all diets show a high dispersion of data at N5 and AD instars.

**Figure 6:**
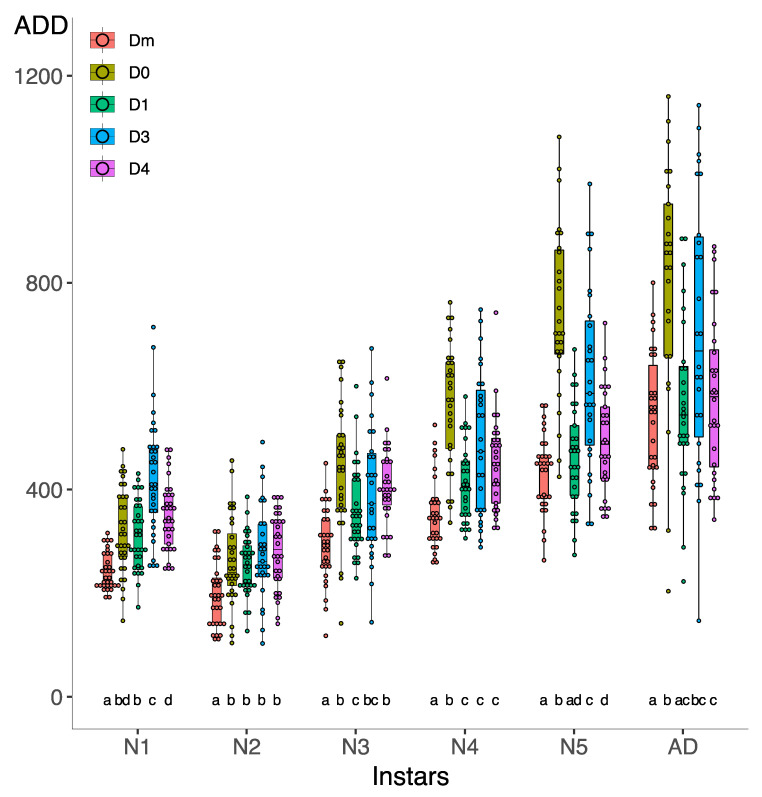
Effect of D0–D1–D3–D4–Dm diets on ADD for each *Zelus renardii* post-embryonic instar in the second generation. Abbreviations: N1 = first instar; N2 = second instar; N3 = third instar; N4 = fourth instar; N5 = fifth instar; AD = adult; ADDs = accumulated degree-days. Lowercase letters indicate significant difference between diets in each instar.

Although there were no significant differences between cumulative ADD per instar between generations, the diets in the second generation (Figure 6) showed different behaviors in each instar compared to the first one.

The N1 (Figure 6) accumulated more ADD than Dm in all the artificial diets tested, especially D3. At N2, N3, and N4, all the artificial diets showed a higher ADD value than Dm. In general, D0 accumulated most ADDs in N2–N4 instars. At N5 and AD, we did not find significant differences between Dm and D1. D0 was the diet with the greatest dispersion of data. Furthermore, as in the first generation, the dispersion of data, especially in the N5 and the AD, is notable for each treatment.

### 3.4. Reproductive Parameters

**Figure 7:**
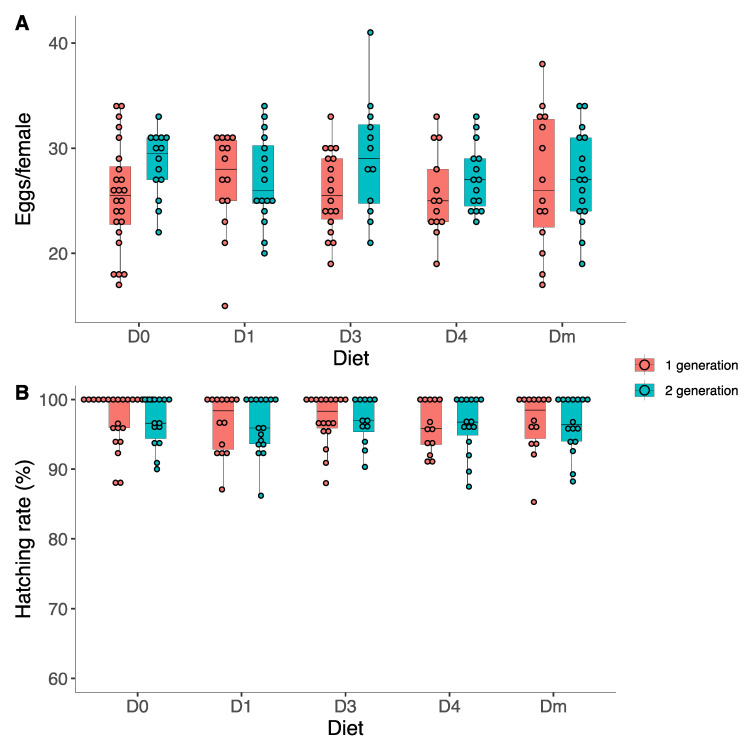
(**A**) Number of eggs per female of *Zelus renardii*, recorded per D0–D1–D3–D4–Dm diets in the first (red plots) and second generations (blue plots) and (**B**) hatching rate of *Zelus renardii* egg-masses (%) recorded per each diet.

The mean number of eggs per female in the first generation was 26.04 ± 4.83 (Figure 7A). LABs fed *D. melanogaster* had the highest number of eggs per female (26.92 ± 6.52 eggs/female), while those fed the D0 had the lowest number (25.45 ± 5.05 eggs/female).

In the second generation, the mean number of eggs per female was slightly higher (27.71 ± 4.12 eggs/female). In this case, individuals on the artificial diet D3 had the highest laying value (29.16 ± 5.57 eggs/female), while those tested for Dm had the lowest value (26.92 ± 6.52 eggs/female). However, significant differences were only found between the generations but not between diets (two-way ANOVA, F_1,146_ = 5.13, *p* = 0.024) (Table S5). In the first generation, the mean number of eggs per female was 26.04 ± 4.83 versus 27.71 ± 4.12 in the second generation.

The egg-hatching percentage was 96.91 ± 3.85% and 96.48 ± 3.73% in the first and second generations, respectively. However, these differences were non-significant (Figure 7B) (Table S6). The hatching value of the eggs was always higher than 96%, regardless of generation or diet.

In the first generation, we recorded the highest hatching rate in individuals fed the D0 (97.52 ± 3.80%), while the lowest was for D4 (96.15 ± 3.59%). In the second generation, individuals treated with the D3 diet had the highest hatching rate (96.92 ± 3.29%), while the lowest was for D1 (96.17 ± 4.09%). However, no significant differences were found between generations or the tested diets.

### 3.5. Life Tables

Table 1, Table 2, Table 3, Table 4 and Table 5 present the LAB actuarial data for each treatment and generation tested.

According to Carey [95], all diets that enabled the completion of post-embryonic development ensured a growing population trend according to the species’ reproductive potential, as predicted by life tables (Table 1, Table 2, Table 3, Table 4 and Table 5). *T* fluctuations between the first and second generations can be ascribed to the different assortment of females between one generation and the next (Figure 2). D0 is the diet with the highest overall *T*, followed by D1, D3, Dm, and D4.

### 3.6. Prediction of the Effect of the Diet in Each Instar

**Figure 8:**
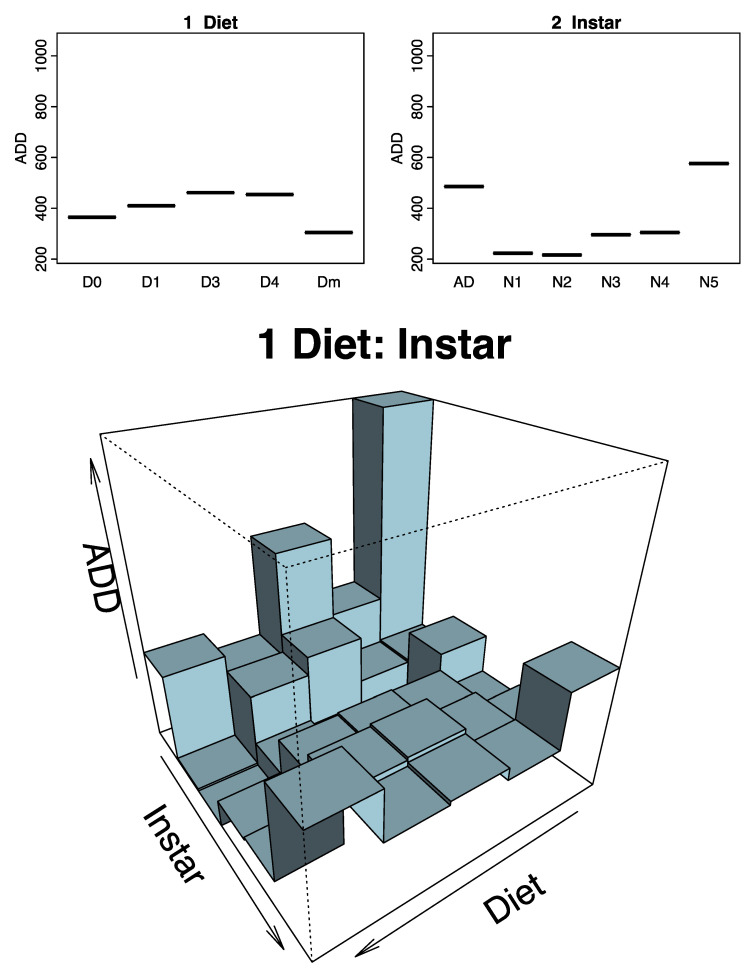
Random Forest plots. The upper plots (1 “diet” and 2 “instar”) indicate expected ADDs based on D0–D1–D3–D4–Dm diets or AD–N1–N2–N3–N4–N5 *Zelus renardii* instars. The lower plot shows expected ADDs based on diet and depending on instar. Abbreviations: N1 = first instar; N2 = second instar; N3 = third instar; N4 = fourth instar; N5 = fifth instar; AD = adult; ADDs = accumulated degree-days.

The predictive model explains 39.5% of the variance with the instar and diet variables. The most important variable for predicting ADD was “instar” (Figure S1). The model predicted a higher ADD value at instar N5 and AD (Figure 8). On the other hand, the instar with the lowest predicted ADD was N1, followed by N2 (Figure 8). Regarding the ADD expected for each diet, Dm would require the lowest number of ADD, closely followed by D0 and D1. In contrast, individuals fed with D3 and D4 would accumulate more ADDs.

In the predictions of ADDs (according to diet and instar), we observed an interaction between factors. At instars N1, N2, N3, and N4, D0 would be the most likely Dm. Furthermore, D1, D3, and D4 would accumulate fewer ADDs at N5 instar than D0 or Dm. Finally, in the case of Ad, D4 and D1 collect less ADD than Dm. Nevertheless, the rest of the artificial diets would allow insects to accumulate more ADDs, especially D0.

**Figure 9:**
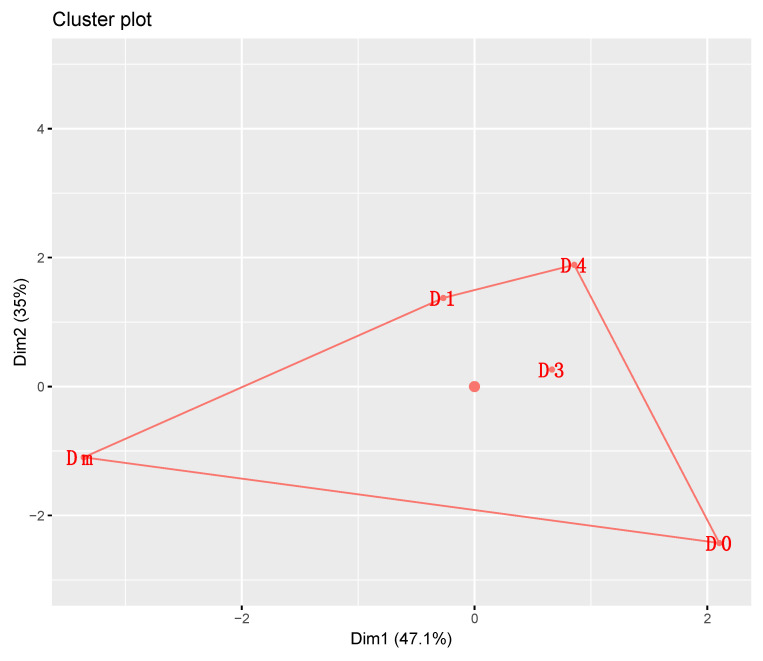
K-means clustering analysis of the different parameters (sex ratio, mortality rate, ADD accumulation, number of eggs/female, and hatching rate) of the *Zelus renardii* individuals fed with the living prey (Dm) and artificial diets (D0, D1, D3, D4). Abbreviations: ADDs = accumulated degree-days; Dm = *Drosophila melanogaster*.

The unsupervised K-means analysis explained 82.1% of the model’s variance (Figure 9). With the Gap statistic method, the separation into one cluster was estimated to be optimal (Figure S2). With this method, there was no evidence that the characteristics of the individuals fed with the artificial diets differed from those presented in those fed with Dm (Table S7).

## 4. Discussion

Under laboratory conditions, LAB has demonstrated a promising predatory capacity against *Xylella* vectors and other olive pests, enhancing its suitability as a biocontrol agent in olive orchards.

The mass production of predators represents a crucial precondition for their use in biocontrol programs. Few predatory reduviids are commercially available for pest biocontrol [27]. Until now, there was no information regarding the mass-rearing of *Z. renardii* using artificial diets.

Mass-rearing success depends on the quality of the artificial diet [27] and the species’ propensity to live in captivity. LAB adapts well to rearing with live prey [47] and oligidic, meridic, and holidic artificial formulations. The LABs completed their egg-to-egg life cycle twice with all the proposed formulations except for D2. The diets were palatable, stable, nutritional, and bioavailable for LABs, such that they completed two subsequent life cycles as expected from an effective artificial diet formulation [5].

The diets D0, D1, D3, and D4 allowed for the LAB rearing in captivity for two generations. The D1 formulation showed cumulative performances like Dm. A successful combination of species-specific diet and rearing technique will allow testing different options for using *Z. renardii* as a biocontrol. Furthermore, the performance of the diets for ADDs differed concerning the LAB instars, indicating that nutritional requirements could be instar-specific. The correct content of nutrients in the diet is related to many factors, such as body size, immunity, and survival [96,97,98,99,100,101,102,103,104,105,106,107,108]. In future investigations, to improve growth performance, body development, and survival, deepening the protein composition of diets, regarding the aminoacidic and fat composition, and identifying its acid profile will be appropriate.

Generally, the mass-rearing of insects in captivity intrinsically imposes a change in the genetic structure of the reared species [10], exerting selective pressure on the cohort. Mass rearing could influence sex ratios, but little knowledge exists about the factors influencing sex determination in Reduviidae [27,82,109]. Adult LABs reared on Dm, D1, D3, and D4 maintained a sex ratio of approximately 1:1 in both generations. Only in the first generation did significant differences in the sex ratio of adults reared on D0 (17% ♂♂ and 83% ♀♀). In the genus *Zelus*, sexual determinism depends on the karyotype of the embryo [109] and is not influenced by environmental conditions. Moreover, in the second generation, D0 did not show any differences in sex ratio. Thus, the differences observed in the D0 sex ratio between generations may depend on the random choice of newborn individuals forming the first cohort.

Having abundant individuals in one or more cohorts will allow us to test the efficacy of LABs against other prey-size classes, increasing the predator’s efficacy knowledge. Similarly, several mixed LAB cohorts of different instars can achieve prey size-dependent predation on other sympatric target pests. We know that young LABs prey on *Drosophila* spp. or *Megaselia* spp. larvae and LAB adults mostly ignore these small insects but quickly subdue *P. spumarius* and *B. oleae* [47]. These observations suggest using different LAB age cohorts in olive or fruit orchard management. A diet that maximizes LAB’s fitness will allow maximum LAB efficacy for inundation against target prey, such as Xf vectors and similar hemipterans.

However, an abundant population of Reduviidae will better tolerate intensive treatments during selection and sanitization to start its mass rearing, leaving alive a proper number of individuals for selection. The sanitization of LABs, in turn, will result in a reasonable basis for the subsequent selection of LAB lines.

Unfortunately, we do not have substances to aggregate or maintain in an area population of LAB effective to pests’ biocontrol despite LAB is sensitive to manipulation by VOCs or other semiochemicals. By the time, we cannot suggest LABs in inoculative or pre-emptive biocontrol actions for the same reason [110,111,112,113].

ADDs or GDDs (growing degree-days) accurately predict insect development based on heat accumulation [114]. Each insect requires specific heat accumulation to carry out post-embryonic development; DDs (degree-days) interpret this accumulation. ADDs are primarily used in forensic entomology [115,116,117] and predictive models for controlling plant pests [114,118,119,120]. The use of ADDs in mass rearing allows for predicting the time required to complete post-embryonic development, regardless of the season in which rearing occurs, opening the prospect of continuous annual rearing. With a constant temperature and by using eggs as a biofix (starting point for ADDs), it was possible to predict the duration of LAB’s cycle in captivity (estimated ADDs per stage shown in Table 1, Table 2, Table 3, Table 4 and Table 5). With an average daily temperature of 25° C, LABs accumulated enough ADDs to produce between two and three generations/year in captivity, as it occurs in nature [33].

## 5. Conclusions

The recent entrance of *Zelus renardii* in the Palaearctic has opened a promising avenue for the biocontrol of pests. The successful rearing of LAB with artificial diets for two successive generations suggests the feasibility of LAB mass rearing and testing the predator in field small-scale experiments. Four formulations sustain the LAB captive post-embryonic development. Meridic D4, holidic D1–D3, and oligidic D0 diets offer higher trends, presuming the opportunity to use LABs in biocontrol actions. D2 does not allow LAB post-embryonic development. Diets require further delve concerning predator and developmental stage-specific requirements. 

Further developments will include optimizing the diet delivery formulation and customization. ADDs, in combination with cohort rearing, forecast the time to complete post-embryonic development. Diets allow two or three generations per year, aligned with the feral population.

Still, no evidence exists of LAB’s negative ecological impact, whose population may be self-regulated through cannibalism. The opportunity to manipulate *Z. renardii* with semiochemicals and future better-performing diets will offer insights for reduviid use in pest management.

## Figures and Tables

**Table 1:** Life table of the two cohorts of 33 *Zelus renardii* reared with Dm (*Drosophila melanogaster*). Abbreviations: EG = egg; N1 = first instar; N2 = second instar; N3 = third instar; N4 = fourth instar; N5 = fifth instar; AD = adult; T_1_ = population trend of 1st generation; T_2_ = population trend of 2nd generation; T_Dm_ = population trend of *Z. renardii* reared with Dm.

Dm						
Instar (x)	Number Living (N)	Fraction Surviving (lx)	Period Survival (px)	Period Mortality (qx)	Frequence of Deaths (dx)	Expectation of Life (ADD) (ex)
First generation						
EG	33	1	1	0	0	2527.5
N1	33	1	1	0	0	2399.5
N2	33	1	1	0	0	2137.1
N3	33	1	0.97	0.03	0.03	1892.3
N4	32	0.97	0.97	0.03	0.03	1558.5
N5	31	0.94	0.94	0.06	0.06	1128.7
AD	29	0.88	0	1	0.88	489.3
EG	338					
T_1_ = 10.24						
Second generation						
EG	33	1	1	0	0	2184.2
N1	33	1	0.97	0.03	0	2056.2
N2	32	0.97	0.97	0.03	0.03	1814.4
N3	31	0.94	0.97	0.03	0.03	1616.5
N4	30	0.91	1	0	0	1323.4
N5	30	0.91	1	0	0	969.2
AD	30	0.91	0	1	0.91	537.3
EG	416					
T_2_ = 12.61						
T_Dm_ = 11.43						

**Table 2:** Life table of the two cohorts of 33 *Zelus renardii* reared with D0 (beef liver and egg yolk-based oligidic diet). Abbreviations: EG = egg; N1 = first instar; N2 = second instar; N3 = third instar; N4 = fourth instar; N5 = fifth instar; AD = adult; T_1_ = population trend of 1st generation; T_2_ = population trend of 2nd generation; T_D0_ = population trend of *Z. renardii* reared with D0.

D0						
Instar (x)	Number Living (N)	Fraction Surviving (lx)	Period Survival (px)	Period Mortality (qx)	Frequence of Deaths (dx)	Expectation of Life (ADD) (ex)
First generation						
EG	33	1	1	0	0	3552.2
N1	33	1	0.97	0.03	0.03	3424.2
N2	32	0.97	1	0	0	3039.7
N3	32	0.97	0.97	0.03	0.03	2437.6
N4	31	0.94	0.94	0.06	0.06	2117.5
N5	29	0.88	0.93	0.07	0.06	1860.4
AD	27	0.82	0	1	0.82	1266
EG	624					
T_1_ = 18.91						
Second generation						
EG	33	1	1	0	0	3300.1
N1	32	0.97	1	0	0	3172.1
N2	32	0.97	0.97	0.03	0.03	2849.8
N3	31	0.94	0.94	0.06	0.06	2587.0
N4	29	0.88	0.93	0.07	0.06	2143.9
N5	27	0.82	1	0	0	1580.0
AD	27	0.82	0	1	0.82	838.4
EG	378					
T_2_ = 11.45						
T_D0_ = 15.18						

**Table 3:** Life table of the two cohorts of 33 *Zelus renardii* reared with D1 (holidic diet based on Meritene MOBILIS^®^). Abbreviations: EG = egg; N1 = first instar; N2 = second instar; N3 = third instar; N4 = fourth instar; N5 = fifth instar; AD = adult; T_1_ = population trend of 1st generation; T_2_ = population trend of 2nd generation; T_D1_ = population trend of *Z. renardii* reared with D1.

D1						
Instar (x)	Number Living (N)	Fraction Surviving (lx)	Period Survival (px)	Period Mortality (qx)	Frequence of Deaths (dx)	Expectation of Life (ADD) (ex)
First generation						
EG	33	1	1	0	0	2647.6
N1	33	1	0.94	0.06	0.06	2519.6
N2	31	0.94	1	0	0	2158.4
N3	31	0.94	0.94	0.06	0.06	1907.2
N4	29	0.88	0.97	0.03	0.03	1476.7
N5	28	0.85	1	0	0	1038.2
AD	28	0.85	0	1	0.85	593.9
EG	364					
T_1_ = 11.03						
Second generation						
EG	33	1	1	0	0	2495.6
N1	33	1	0.97	0.03	0.03	2367.6
N2	32	0.97	0.97	0.03	0.03	2056.6
N3	31	0.94	0.97	0.03	0.03	1802.1
N4	30	0.91	0.97	0.03	0.03	1442.7
N5	29	0.88	0.97	0.03	0.03	1028.6
AD	28	0.85	0	1	0.85	566.4
EG	432					
T_2_ = 13.09						
T_D1_ = 12.06						

**Table 4:** Life table of the two cohorts of 33 *Zelus renardii* reared with D3 (holidic artificial diet based on Nidina^®^ 2 OPTIPRO^®^). Abbreviations: EG = egg; N1 = first instar; N2 = second instar; N3 = third instar; N4 = fourth instar; N5 = fifth instar; AD = adult; T_1_ = population trend of 1st generation; T_2_ = population trend of 2nd generation; T_D3_ = population trend of *Z. renardii* reared with D3.

D3						
Instar (x)	Number Living (N)	Fraction Surviving (lx)	Period Survival (px)	Period Mortality (qx)	Frequence of Deaths (dx)	Expectation of Life (ADD) (ex)
First generation						
EG	33	1	1	0	0	3028.7
N1	33	1	0.97	0.03	0.03	2900.7
N2	32	0.97	0.97	0.03	0.03	2334.6
N3	31	0.94	0.94	0.06	0.06	2052.8
N4	29	0.88	0.93	0.07	0.06	1668.8
N5	27	0.82	1	0	0	1282
AD	27	0.82	0	1	0.82	842.8
EG	468					
T_1_ = 14.18						
Second generation						
EG	33	1	1	0	0	3034.1
N1	33	1	0.88	0.12	0.12	2906.1
N2	29	0.88	0.93	0.07	0.06	2479.9
N3	27	0.82	1	0	0	2199.3
N4	27	0.82	1	0	0	1805.4
N5	27	0.82	0.96	0.04	0.03	1317.5
AD	26	0.79	0	1	0.79	702.3
EG	324					
T_2_ = 9.82						
T_D3_ = 12						

**Table 5:** Life table of the two cohorts of 33 *Zelus renardii* reared with D4 (meridic artificial diet based on Meritene MOBILIS^®^, KCl, and OB). Abbreviations: EG = egg; N1 = first instar; N2 = second instar; N3 = third instar; N4 = fourth instar; N5 = fifth instar; AD = adult; T_1_ = population trend of 1st generation; T_2_ = population trend of 2nd generation; T_D4_ = population trend of *Z. renardii* reared with D4.

D4						
Instar (x)	Number Living (N)	Fraction Surviving (lx)	Period Survival (px)	Period Mortality (qx)	Frequence of Deaths (dx)	Expectation of Life (ADD) (ex)
First generation						
EG	33	1	1	0	0	2760.1
N1	33	1	0.94	0.06	0.06	2632.1
N2	31	0.94	0.94	0.06	0.06	2054.2
N3	29	0.88	0.97	0.03	0.03	1736.4
N4	28	0.85	0.96	0.04	0.03	1337.3
N5	27	0.82	1	0	0	901.4
AD	27	0.82	0	1	0.82	492.1
EG	338					
T_1_ = 10.24						
Second generation						
EG	33	1	0	0	0	2690.9
N1	33	1	0.97	0.03	0.03	2562.9
N2	32	0.97	0.94	0.06	0.06	2213.0
N3	30	0.91	0.97	0.03	0.03	1931.2
N4	29	0.88	1	0	0	1523.5
N5	29	0.88	0.97	0.03	0.03	1071.1
AD	28	0.85	0	1	0.85	578.9
EG	390					
T_2_ = 11.82						
T_D4_ = 11.03						

## Data Availability

The authors can share the references collected for this contribution.

## Supplementary Materials

The following supporting information can be downloaded at https://www.mdpi.com/article/10.3390/insects15080607/s1, Table S1: Ingredients and nutritional information of Meritene^®^ MOBILIS^®^ (Nestlé^®^ Health Science, Nestlé S.A., Vevey, Switzerland); Table S2: Ingredients and nutritional information of NIDINA^®^ OPTIPRO^®^ 2 (Nestlé^®^ Baby&Me, Nestlé S.A., Vevey, Switzerland); Table S3 Welch’s ANOVA of ADD of the 1st generation with diet as a fixed factor. Abbreviation: Df = degree of freedom; Table S4: Welch’s ANOVA of ADD of the 2nd generation with diet as a fixed factor. Abbreviation: Df = degree of freedom; Table S5: Two-way ANOVA of eggs/female between generations and diets. Abbreviations: Df = degree of freedom; Sum sq = sum of square; Mean sq = mean square; Table S6: Two-way ANOVA of eggs hatching between generations and diets. Abbreviations: Df = degree of freedom; Sum sq = sum of square; Mean sq = mean square; Table S7: Summary of the characteristics of insects treated with the different diets. Abbreviations: ADD = accumulated degree-days; N1 = first instar; N2 = second instar; N3 = third instar; N4 = fourth instar; N5 = fifth instar; AD = adult; Figure S1: Dotchart of variable importance as measured by a Random Forest; Figure S2: Optimal number of clusters in the k-means analysis according to Gap Statistic.
