# Supplementary material for: Performance of Artificial Diets for Zelus renardii (Hemiptera: Reduviidae) Rearing
Source: Insects. 2024 Aug 12;15(8):607. doi: 10.3390/insects15080607 (PMC11354418; doi:10.3390/insects15080607)
Supplement: Supplementary file 1 [file insects-15-00607-s001.zip › insects-3100810-supplementary.pdf]

Supplementary materials

Table S1: Ingredients and nutritional information of Meritene® MOBILIS® (Nestlé® Health Science, Nestlé S.A., Vevey, Switzerland).

Ingredients					
Skimmed milk, milk protein, maltodextrin, inulin, minerals (calcium carbonate, magnesium carbonate, magnesium citrate, zinc gluconate, iron phosphate, calcium phosphate, copper gluconate, manganese sulphate, sodium selenite), sunflower oil, vitamins (C, E, niacin, pantothenic acid, B1, B6, A, B2, folic acid, K, biotin, D, B12), emulsifier: soy lecithin.					
**Nutrients**	**Unit**	**100g of Meritene® MOBILIS®**	**Nutrients**	**Unit**	**100g of Meritene® MOBILIS®**
total fat	g	3.5	**Minerals**		
carbohydrates	g	36.5	potassium	mg	800
fiber	g	24	chloride	mg	500
proteins	g	44.8	calcium	mg	950
salt (=Na(g) x 2.5)	g	1	phophorous	mg	510
**Vitamins**			magnesium	mg	250
vit. A	µg	590	iron	mg	6
vit. D	µg	23	zinc	mg	6.7
vit. E	mg	10	copper	mg	0.62
vit. K	µg	70	manganese	mg	1.1
vit. C	mg	180	selenium	µg	41
vit. B1	mg	1.4			
vit. B2	mg	1			
vit. B6	mg	1.4			
folic acid	µg	170			
vit. B12	µg	3.9			
biotin	µg	39			
pantothenic acid	µg	4.4			

Table S2: Ingredients and nutritional information of NIDINA® OPTIPRO® 2 (Nestlé® Baby&Me, Nestlé S.A., Vevey, Switzerland).

**Ingredients**
Skimmed milk, partially demineralised and fractionated whey powder, vegetable oils (palm, rapeseed, coconut, sunflower), maltodextrin, lactose, soluble fibres (galacto-oligosaccharides from milk and fructo-oligosaccharides), minerals (calcium, potassium, sodium, magnesium, iron, zinc, copper, iodine, selenium), emulsifier: soy lecithin, vitamins (C, E, pantothenic acid, niacin, A, B1, B6, riboflavin, folic acid, biotin, D,K, B12), whey protein, *L. reuteri*

**Nutrients**	**Unit**	**100g of Nidina® 2 OPTIPRO®**	**Nutrients**	**Unit**	**100g of Nidina® 2 OPTIPRO®**
total fat	g	23.6	**Minerals**		
carbohydrates	g	59	sodium	mg	190
fiber	g	2.9	potassium	mg	550
proteins	g	9.6	chloride	mg	360
salt (=Na(g) x 2.5)	g	0.48	calcium	mg	560
**Vitamins**			phophorous	mg	310
vit. A	µg	530	magnesium	mg	50
vit. D	µg	9	iron	mg	7.3
vit. E	mg	9.3	zinc	mg	5.2
vit. K	µg	46	copper	mg	0.35
vit. C	mg	85	manganese	µg	50
thiamine	mg	1	fluoride	µg	<60
riboflavin	mg	1.2	selenium	µg	8.6
niacin	mg	4.4	iodine	µg	13
vit. B6	mg	0.54	**Other nutrients**		
folic acid	µg	118	linoleic acid	mg	3800
vit. B12	µg	1.2	α-linolenic acid	mg	460
biotin	µg	16	lactose	g	35.5
pantotenic acid	mg	5.2			

Table S3 Welch's ANOVA of ADD of the 1st generation with Diet as a fixed factor. Abbreviation: Df = degree of freedom

Factor	Df	*F value*	*p-value*
Diet	4	5.16	0.0111*
Residuals	62.29		

Table S4: Welch's ANOVA of ADD of the 2nd generation with Diet as a fixed factor. Abbreviation: Df = degree of freedom

Factor	Df	*F value*	*p-value*
Diet	4	41.70	<0.001
Residuals	65.55		

Table S5: Two-way ANOVA of eggs/female between generations and diets. Abbreviations: Df = degree of freedom; Sum sq = sum of square; Mean sq = mean square

Factor	Df	Sum sq	Mean sq	*F value*	*p-value*
Generation (G)	1	107.6	107.56	5.13	0.024
Diet (D)	4	14.3	3.57	0.17	0.953
G x D	4	69.0	17.24	0.82	0.516
Residual	146	3059.5	20.96		

Table S6: Two-way ANOVA of eggs hatching between generations and diets. Abbreviations: Df = degree of freedom; Sum sq = sum of square; Mean sq = mean square

Factor	Df	Sum sq	Mean sq	*F value*	*p-value*
Generation (G)	1	7.400	7.392	0.492	0.484
Diet (D)	4	22.000	5.506	0.367	0.832
G x D	4	4.200	1.047	0.070	0.991
Residual	146	2192.600	15.018		

Table S7: Summary of the characteristics of insects treated with the different diets. Abbreviations: ADD = Accumulated Degree-Days; N1 = first instar; N2 = second instar; N3 = third instar; N4 = fourth instar; N5 = fifth instar; Ad = adult

Diet	ADD_N1	ADD_N2	ADD_N3	ADD_N4	ADD_N5	ADD_AD	female_ratio (%)	mortality_ratio (%)	Hatching rate (%)	Egg/females
Dm	224.43	210.36	294.39	330.77	512.14	473.59	0.49	0.07	96.53	27.06
D0	342.47	425.8	372.87	380.01	600.98	916.45	0.67	0.18	97.22	26.57
D1	339.73	248.92	382.59	405.45	420.088	532.57	0.53	0.15	96.31	26.93
D3	496.13	263.92	352.17	393.41	438.24	683.6	0.56	0.19	97.07	27.16
D4	463.87	291.11	383.91	415.49	422.08	491.58	0.51	0.16	96.25	26.42

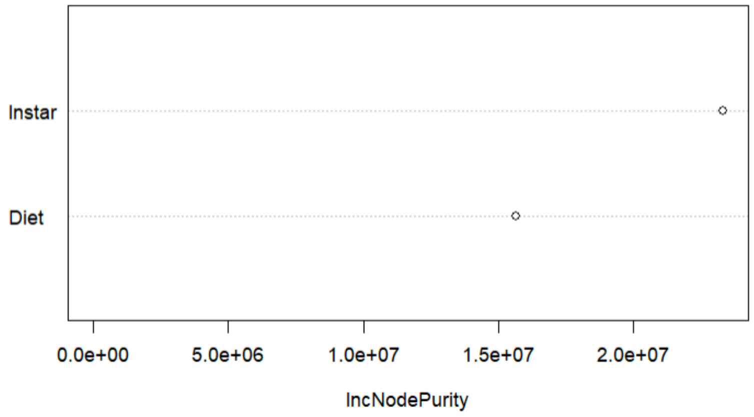

Figure S1: Dotchart of variable importance as measured by a Random Forest

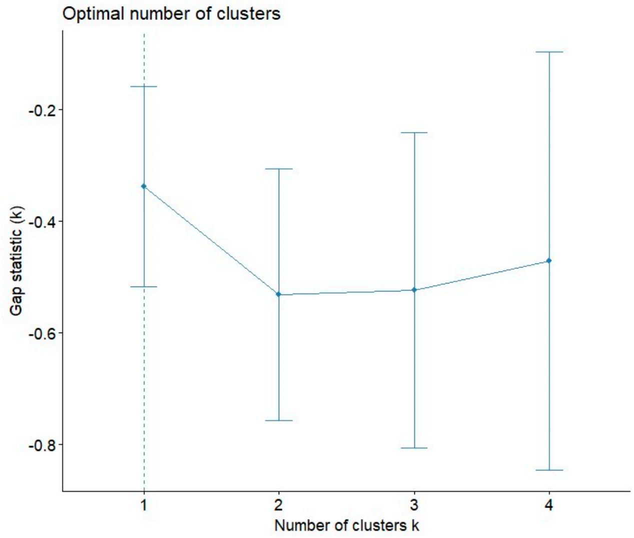

Figure S2: Optimal number of clusters in the k-means analysis according to Gap Statistic
